# Endobronchial ultrasound‐guided cryobiopsy for diagnosing a case of granulomatosis with polyangiitis

**DOI:** 10.1002/rcr2.1385

**Published:** 2024-05-15

**Authors:** Teresa Calari, Simone Petrarulo, Alessandra Dubini, Sara Piciucchi, Claudia Ravaglia, Venerino Poletti

**Affiliations:** ^1^ Department of Medical and Surgical Sciences (DIMEC) University of Bologna Bologna Italy; ^2^ Respiratory and Critical Care Unit IRCCS Azienda Ospedaliero‐Universitaria di Bologna Bologna Italy; ^3^ Department of Medical Specialities, Pulmonology Unit GB Morgagni—L. Pierantoni Hospital Forlì Italy; ^4^ Department of Pathology GB Morgagni—L. Pierantoni Hospital Forlì Italy; ^5^ Department of Radiology GB Morgagni—L. Pierantoni Hospital Forlì Italy; ^6^ Department of Medical and Surgical Sciences (DIMEC) University of Bologna/Forlì Campus Forlì Italy; ^7^ Department of Respiratory Diseases and Allergy Aarhus University Hospital Aarhus Denmark

**Keywords:** EBUS‐guided cryobiopsy, EBUS‐TBNA, granulomatosis with polyangiitis, mediastinal cryobiopsy, vasculitis

## Abstract

EBUS‐TBNA has represented a revolution in the diagnosis of intrathoracic pathologies, particularly in lung cancer staging, replacing more invasive methods such as mediastinoscopy. However, its role in diagnosing rare benign or malignant mediastinal disorders is still a matter of debate. Over the past few years, the role of EBUS‐guided cryobiopsy has been increasingly emerging as an innovative and minimally invasive technique in diagnosing these disorders, with an excellent safety profile. In this case report, we present the case of a young man brought to our attention after already undergoing a non‐diagnostic trans thoracic needle aspiration (TTNA) procedure for lung consolidations. In our department, he underwent an initial EBUS‐TBNA procedure with inconclusive rapid on‐site evaluation (ROSE), leading to the decision to perform an EBUS‐guided cryobiopsy, which yielded a diagnosis of granulomatosis with polyangiitis without complications. This clinical case demonstrates that in specific contexts, EBUS‐cryobiopsy represents an excellent diagnostic tool.

## Graphical abstract

This clinical case demonstrates that in specific contexts, EBUS‐cryobiopsy represents an excellent diagnostic tool. A young man was brought to our attention after already undergoing a non‐diagnostic trans thoracic needle aspiration (TTNA) procedure for lung consolidations. In our department, he underwent an initial EBUS‐TBNA procedure with inconclusive rapid on‐site evaluation (ROSE), leading to the decision to perform an EBUS‐guided cryobiopsy, which yielded a diagnosis of granulomatosis with polyangiitis without complications.

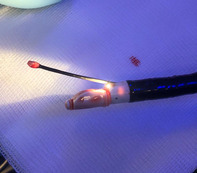

## INTRODUCTION

Granulomatosis with polyangiitis (GPA) is a rare and severe necrotizing vasculitis affecting small to medium‐sized vessels, within the spectrum of antineutrophil cytoplasmic antibodies (ANCA)‐associated vasculitides (AAV). GPA typically manifests in the upper and lower respiratory tracts and kidneys, leading to significant morbidity and mortality. Histologically, it presents in the lungs with geographic necrosis, inflammatory background containing giant cells and vasculitis. One of its hallmark features is the presence of ANCAs–cytoplasmic in around 90% of systemic cases–targeting proteinase 3 (PR3), a serine protease found in neutrophil granules. Guidelines recommend biopsies to aid in establishing a diagnosis, with surgical lung biopsy considered the standard method. Regular transbronchial biopsy has a low diagnostic yield (30%).^1^ There is currently no data regarding the use of cryobiopsy for achieving a confident diagnosis of AAV.

## CASE REPORT

**FIGURE 1:**
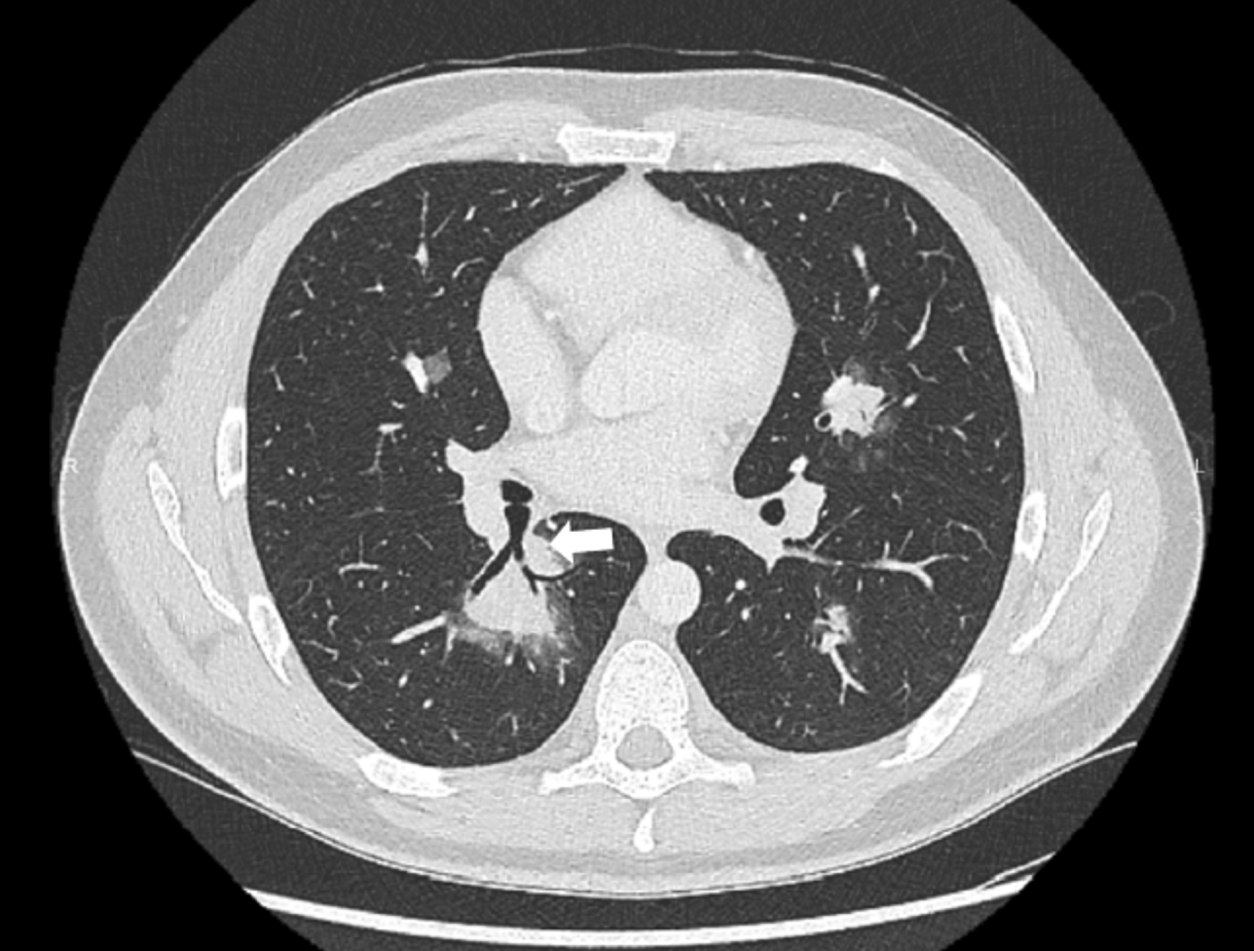
Chest CT scan showing multiple consolidation with peripheral halo sign. The white arrow indicates the target of EBUS‐TBNA and EBUS guided cryobiopsy.

**FIGURE 2:**
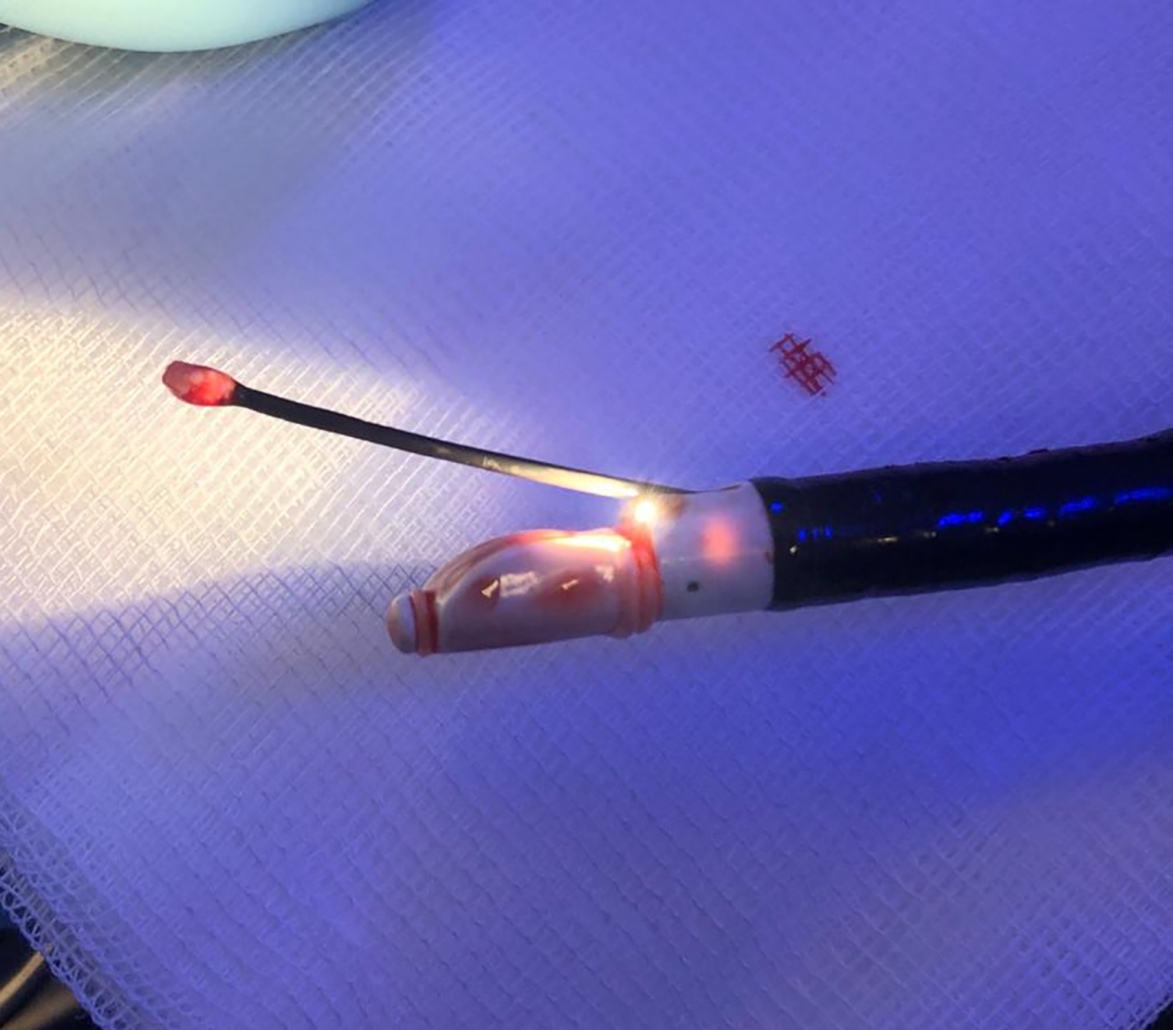
EBUS scope along with one cryobiopsy sample, in which the frozen sample at the probe is visible.

**FIGURE 3:**
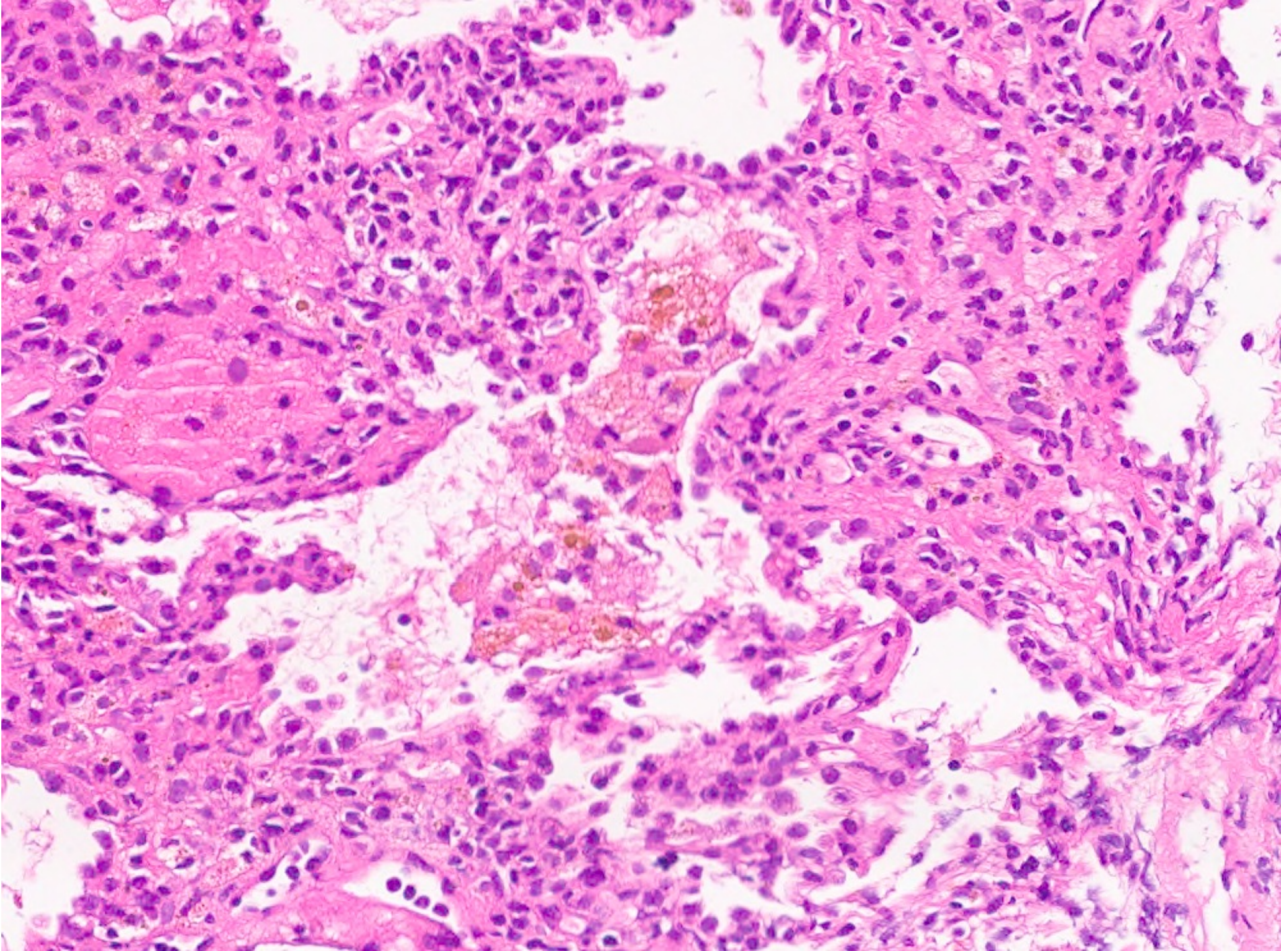
EBUS guided cryobiopsy (staining with H&E) showing capillaritis, alveolar haemorrhage and some giant cells.

Herein, we present a case of a 24‐year‐old man, a nonsmoker and student, with no significant medical history. In December 2023, he experienced persistent thoracic pain accompanied by hemoptysis, without fever or productive cough. Despite empirical antibiotic treatment (cefixime and azithromycin), the patient failed to improve and was subsequently admitted to the local hospital. A chest CT scan revealed bilateral perihilar pseudonodular consolidations with a maximum diameter of 30 mm, partial air bronchogram with faint contrast enhancement, and peripheral halo sign (Figure 1). ANCAs directed against PR3 were positive, while QuantiFERON test, beta‐D‐glucan, and serum galactomannan were negative. Broncho‐Alveolar Lavage (BAL) was performed in the middle lobar bronchus; however, the macroscopic examination did not reveal alveolar haemorrhage, microbiological tests were negative, and cytofluorimetry was within normal limits. High‐dose systemic steroid therapy was initiated, but the patient continued to experience hemoptysis and thoracic pain after 2 weeks. A repeat chest CT scan performed 21 days later showed no change. Consequently, a trans thoracic needle aspiration (TTNA) was performed in the local hospital on a parenchymal consolidation located in the right lower lobe. However, histological examination yielded inconclusive results. Subsequently, the patient was referred to our Department in February 2024 for further investigations. Laboratory tests revealed positivity for anti‐PR3 and anti‐PM‐scl‐75 antibodies, with normal levels of C‐reactive protein and parameters within the normal range for complete blood count and renal function. The patient underwent rigid bronchoscopy (Karl Storz GmbH, Tuttlingen, Germany) under general anaesthesia and endobronchial ultrasound (BF‐UC180F; Olympus Medical Systems, Japan). Subsequently, 5 passes of endobronchial ultrasound transbronchial needle aspiration (EBUS‐TBNA) was performed using a 19‐gauge needle (Olympus Medical Systems, Japan) on a hypoechoic lesion located dorsally to the origin of the right lower lobe bronchus. No significant vascular structure was demonstrated using endosonographic colour‐Doppler imaging. Following the initial puncture with the 19G TBNA needle, EBUS‐guided cryobiopsy was immediately conducted. A 1.1 mm cryoprobe (Erbecryo, Tubingen, Germany) was inserted into the working channel of the EBUS bronchoscope and guided to the targeted site, advancing through the same puncture site created by the 19G TBNA needle under real‐time ultrasound guidance with a freezing time of 6 s (three samples). At the end of each sampling, the linear EBUS was withdrawn along with the cryoprobe through the rigid tracheoscope (Figure 2). No complications occurred during and after the procedure. The pulmonologist rapid onset evaluation (ROSE) of EBUS‐TBNA was inconclusive and histological examination of the EBUS‐TBNA cell block revealed hematic material containing bronchial columnar cells and aggregates of hemosiderin‐laden macrophages with foamy cytoplasm. Conversely, the examination of the cryobiopsy demonstrated alveolated tissue with capillaritis characterized by the presence of neutrophils and eosinophils, along with alveolar septa, hemosiderin‐laden macrophages, and fibrin exudates (Figure 3). Additionally, epithelioid histiocyte aggregates with a granulomatous appearance and rare foci of classical organizing pneumonia were observed in some areas. Overall, these morphological findings were consistent with GPA. Following confirmation of the diagnosis, immunosuppressive therapy with rituximab was initiated.

## DISCUSSION

Nowadays, the interest in the use of cryotechniques combined with EBUS is constantly growing worldwide. This is mainly because of the need to obtain larger tissue samples for histological and molecular analysis. Several studies in the recent literature have already demonstrated the superiority of EBUS guided cryobiopsy over EBUS‐TBNA in terms of diagnostic yield for benign and lymphoproliferative diseases, with significantly low complication rate.^2^, ^3^, ^4^, ^5^ The most common adverse event is minor airway bleeding with no need for intervention, while the reported incidence of grade 3–4 bleeding is 2%.^2^, ^3^ Therefore, in the case we presented, this technique appeared particularly indicated for several reasons such as: the central location of the consolidations, the visibility of the target on endobronchial ultrasound, the inconclusive ROSE, and the absence of vascular structures that could make the biopsy dangerous. In addition, in this case we considered surgical lung biopsy particularly complex to be performed due to the central location of the lesions. To the best of our knowledge, this is the first case of GPA diagnosed using EBUS guided cryobiopsy. In conclusion, we believe that EBUS‐guided cryobiopsy is an effective and highly safe technique which has the potential to avoid non diagnostic procedures, while still maintaining an excellent safety profile. In the future, prospective studies would be useful to further validate the use of this technique for this group of diseases.

## AUTHOR CONTRIBUTIONS

**TC**, **SPe**, **AD**, **SPi**, **CR**: Conception of the manuscript; literature search; drafting of the manuscript. **VP**: Critical review of the manuscript. All the authors approved the final version of the manuscript.

## CONFLICT OF INTEREST STATEMENT

None declared.

## ETHICS STATEMENT

The authors declare that appropriate written informed consent was obtained for the publication of this manuscript and accompanying images.

## Data Availability

Data sharing not applicable to this article as no datasets were generated or analysed during the current study.
